# Alcoholic liver disease in relation to cancer incidence and mortality: Findings from a large, matched cohort study in South Korea

**DOI:** 10.1002/cam4.5614

**Published:** 2023-01-18

**Authors:** Thi Phuong Thao Tran, Minji Han, Ngoc Minh Luu, Jin‐Kyoung Oh

**Affiliations:** ^1^ Department of Cancer Control and Population Health National Cancer Center Graduate School of Cancer Science and Policy Goyang‐si South Korea; ^2^ Center for Population Health Sciences Hanoi University of Public Health Hanoi Vietnam; ^3^ Division of Cancer Prevention National Cancer Center Goyang South Korea; ^4^ Hanoi Medical University Hanoi Vietnam

**Keywords:** alcohol assumption, alcoholic liver disease, cancer incidence, cancer mortality

## Abstract

**Aim:**

To estimate the risk of cancer incidence and mortality among patients with alcoholic liver disease in South Korea.

**Methods:**

A matched cohort study was conducted, including 1,042,185 men (alcoholic liver disease cases: 208,437; controls: 833,748) and 100,400 women (alcoholic liver disease cases: 20,080; controls: 80,320), matched for sex, age, smoking, alcohol consumption, and body mass index at a 1:4 ratio. The risk of cancer incidence and mortality in the alcoholic liver disease group was assessed using Cox proportional hazards regression models.

**Results:**

Both men and women with alcoholic liver disease had an elevated risk of all‐cancer and liver cancer incidence and mortality in comparison with the control group. In men, alcoholic liver disease was associated with a significantly higher risk of development of 10 cancer types, including lip, oral cavity, and pharynx; esophagus; liver; gallbladder and biliary tract; pancreas; larynx; lung; kidney; thyroid gland; and leukemia. Subgroup analysis by hepatitis B and C infection showed increased hazard ratios of all cancer incidences and mortality in the alcoholic liver disease group, regardless of hepatitis B or C infection status. In both sexes, a higher number and more years of hospital or clinic visits for alcoholic liver disease were associated with an increased risk of incidence and mortality from all cancers and liver cancer. A more profound dose–response relationship between alcoholic liver disease and alcohol consumption was observed in women than in men.

**Conclusions:**

Our findings emphasize the need for a clinical surveillance program and the early detection of cancer in patients with alcoholic liver disease.

## Graphical abstract

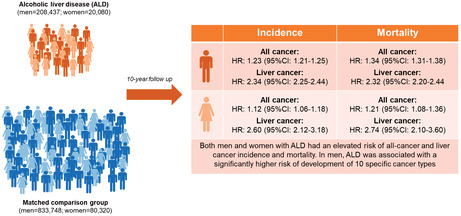

## 1 INTRODUCTION

Alcoholic beverages are classified as group I carcinogens that significantly increase the risk of cancer at various sites.^1^ The process from the initiation of alcohol consumption to cancer development progresses over the long term. Alcohol consumption can lead to disorders and diseases that could play a mediating role in the association between alcohol consumption and cancer risk, owing to sharing the same risk factors (e.g., alcohol consumption and smoking). Alcoholic liver disease (ALD) is the major alcohol‐related chronic disease and the most prevalent chronic liver disease worldwide.^2^, ^3^, ^4^, ^5^ Therefore, together with the primary prevention of cancer by limiting alcohol consumption, data on ALD in relation to cancer could help us appropriately target clinical surveillance and effect the early detection of complications, including cancers.

Although the relationship between alcohol consumption and cancer is well‐documented, our understanding of the association between ALD and cancer is limited. Several cohort studies in European countries have suggested an association between ALD and a higher cancer incidence of not only the liver,^6^, ^7^, ^8^, ^9^, ^10^, ^11^, ^12^ but also other cancer sites including the lung,^6^, ^7^, ^8^, ^9^, ^10^ larynx,^6^, ^7^, ^8^, ^10^ oral cavity,^6^, ^7^, ^8^, ^10^ pharynx,^6^, ^8^, ^10^ esophagus,^8^, ^9^, ^10^ pancreas,^7^, ^8^ kidney,^6^, ^8^ colon,^6^, ^7^, ^8^, ^9^ breast,^6^ and stomach.^8^, ^10^ However, most of these studies were conducted with limited sample size and had missing information on alcohol consumption and/or smoking, which could have confounded the findings. Additionally, scarce information is available regarding the relationship between ALD and mortality due to cancer at other sites. In Asia, research on ALD in relation to cancer is scarce, and most studies have focused on viral B/C hepatitis or cirrhosis,^13^, ^14^, ^15^, ^16^ and non‐alcoholic fatty liver disease.^17^, ^18^

In South Korea, alcohol consumption is a common lifestyle behavior with an increasing trend over the past 40 years^19^; more than 70.5% of men and 51.2% of women drank alcohol (i.e., consumed at least once per month) in 2018.^20^ Consistent with this increase, the prevalence of ALD approximately doubled from 3.8% to 7% from 1998 to 2017,^21^ which could subsequently exacerbate the cancer burden. ALD is estimated to account for 6.8% of the total number of liver cancer cases in South Korea (while hepatitis B and C accounted for 72% and 12.5%, respectively).^19^ For other cancer burdens due to ALD, only one small case–control study suggested that ALD increased the odds of gastric cancer by 5.3 times compared with a control group.^22^ To date, data on the association between ALD and cancer risk in the Korean population have been sparse. Therefore, we aimed to estimate cancer risk in terms of incidence and mortality in patients with ALD, using large‐scale, population‐based cohort study.

## 2 METHODS

### 2.1 Data source

The data for the matched cohort were derived from a National Health Insurance Service (NHIS) database in South Korea. The NHIS is a universal coverage health insurance system covering more than 99% of Korean citizens (nearly 50 million people).^23^ The database includes data on sociodemographics, health examination results, lifestyles and behaviors, and medical diagnoses and treatments; a detailed description of data collection and variables is presented elsewhere.^24^ We extracted a data of NHIS on 8,968,212 people who underwent health screening in 2002 and 2003.

### 2.2 Matched cohort

From January 1, 2002, to December 31, 2008, there were 286,156 incident cases of ALD, based on the International Classification of Diseases, tenth Revision (ICD‐10), code K70. The index date for cases was defined as the first date of diagnosis, with ALD as the primary diagnosis. After excluding individuals who (a) did not attend the general health examination within the 4 years since their K70 diagnosis (including the year of diagnosis; i.e., missing all information on lifestyle behaviors; *n* = 31,720), (b) had missing data on any matched variables (e.g., age, sex, smoking, alcohol consumption, body mass index [BMI]; *n* = 17,985), (c) had cancer before their ALD diagnosis or had cancer within 1 year after their ALD diagnosis (i.e., a 1‐year washout period), to ensure the causal relationship (*n* = 4840), and (d) had errors in cancer incidence or death dates (*n* = 3094), a total of 228,517 ALD cases were finally included in our study. We selected the 2002–2008 period for claims ALD cases because consistent lifestyle behavior‐related variables were obtained in all cases, which was due to changing the general health examination questionnaire in 2009.

**FIGURE 1:**
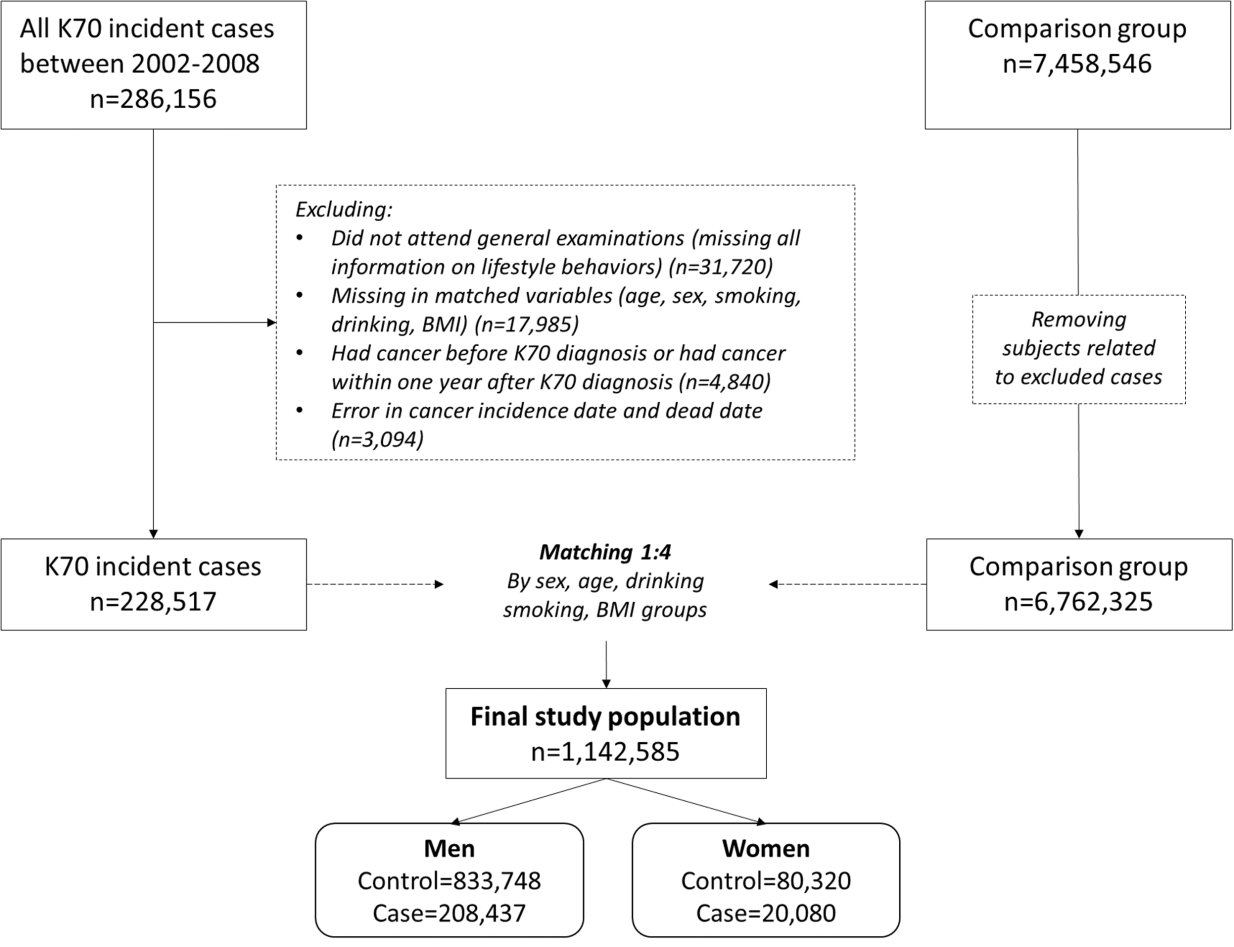
A flow diagram of the study population.

The index date for the comparison group (i.e., non‐ALD individuals) was randomly assigned from January 1, 2002, to December 31, 2008, based on the distribution of the index dates in the case group. After excluding individuals who met the exclusion criteria of the ALD cases, 6,762,325 cancer‐free individuals were included in the comparison group. Subsequently, we matched the case and control groups using propensity score matching with greedy matching—considering index date, age, smoking, alcohol consumption, and BMI group—separately by sex. We matched one ALD patient with four controls. Finally, this study included 1,042,185 men (cases: 208,437; controls: 833,748) and 100,400 women (cases: 20,080; controls: 80,320). Because ALD is known to affect men more often than women,^25^ we matched and analyzed the data separately by sex. A flowchart of the study sample is shown in Figure 1, and the study timeframe is illustrated in Figure S1.

### 2.3 Outcomes and follow‐up

Cancer incidence was identified using the ICD‐10 codes of the primary diagnosis and a special code (V193) for cancer registration in the NHIS database (i.e., indicating a confirmed cancer diagnosis for expanded benefit coverage). Cancer death was obtained from Statistics Korea,^26^ based on the ICD‐10 codes of the causes of death. We determined cancer incidence and mortality using ICD codes: all cancer (C00–C97) and specific cancers, including lip, oral cavity, and pharynx, C00–C14; esophagus, C15; stomach, C16; colon and rectum, C18–C20; liver, C22; gallbladder and biliary tract, C23–C24; pancreas, C25; larynx, C32; lung, C33–C34; breast, C50; cervix uteri, C53; corpus uteri, C54; ovary, C56; prostate, C61; testicular, C62; kidney, C64; bladder, C67; brain, C70–C72; thyroid gland, C73; Hodgkin lymphoma, C81; multiple myeloma and malignant plasma cell neoplasm, C90; leukemia, C91–C95; and non‐Hodgkin lymphoma, C82–C86, C96. The ALD case and control groups were followed up from the index date to the date of onset of cancer, cancer death, death, or the end of follow‐up (December 31, 2018), whichever came first.

### 2.4 Hospital visits due to ALD

Three variables related to hospital visits, including the total number of clinic or hospital visits due to ALD, the total number of years visiting a clinic or hospital for ALD, and the average number of clinic or hospital visits per year for ALD; these were measured to indirectly assess the severity of ALD. The total number of clinic or hospital visits and the total number of years visiting a clinic or hospital were accounted for within the follow‐up period, and categorized into three groups: 0 (control group), 1, and ≥2 visit(s)/year(s). The average number of clinic or hospital visits per year was defined as the total number of clinic or hospital visits for ALD divided by the total number of years visiting a clinic or hospital for ALD. It was grouped as 0 (control group), 1, or ≥1 visit/year.

### 2.5 Covariates

The look‐back period for obtaining data on the covariates was a maximum of 7 years and 4 years before the index date (including the year of the index date) for alcohol consumption and other variables, respectively. Given that ALD develops as a result of long‐term alcohol consumption, we attempted to capture the longest duration of alcohol consumption by indicating its mean value during the look‐back period. Alcohol consumption (ethanol in g/day) was defined as the frequency of alcohol consumption multiplied by the number of alcoholic drinks per day.^27^ The mean alcohol consumption was computed and categorized as <5 g/day, 5–9.9 g/day, 10–19.9 g/day, 20–29.9 g/day, and ≥30 g/day. Data on other covariates were obtained from the most recent year that the data were available within the look‐back period. Data on covariates were extracted from the general health examination questionnaire, including sociodemographic characteristics (age, sex, and income), lifestyle behaviors, disease‐related variables. Smoking status was categorized as never smoked, ex‐smoker, and current smoker. The frequency of physical activity per week included five subgroups: none, 1–2 times, 3–4 times, 5–6 times, and every day. BMI was categorized into three groups: underweight or normal, <23 kg/m^2^; overweight, 23–24.9 kg/m^2^; and obese, ≥25 kg/m^2^.^28^ Hypertension status was classified as normal, systolic <120 mm Hg, and diastolic <80 mm Hg; pre‐hypertension, 120 mm Hg ≤ systolic ≤139 mm Hg or 80 mm Hg ≤ diastolic ≤89 mm Hg; and hypertension, systolic ≥140 mm Hg or diastolic ≥90 mm Hg.^29^ Fasting blood sugar was determined as normal, <100 mg/dl; prediabetes, 100–125 mg/dl; and diabetes, ≥126 mg/dl.^30^ Total cholesterol was measured and grouped as normal, <200 mg/dl; borderline high 200–239 mg/dl; and high, ≥240 mg/dl.^31^ The CCI was also calculated using ICD‐10 codes.^32^ Blood pressure and blood test were measured using certified and calibrated equipment by medical staffs.

### 2.6 Statistical analysis

The characteristics of the study sample are presented as means with standard deviations for age and as frequencies with percentages for categorical variables. Propensity score matching (PROC PSMATCH in SAS software) was used to match cases and controls in a 1:4 ratio. As an insufficient number of controls could be matched to cases, we applied greedy matching rather than exact matching, in which the four nearest control units (non‐ALD) were matched to each treated unit (ALD case), accounting for sex, age, the year of the index event, smoking, alcohol consumption, and BMI group in the matched design. Person‐years were calculated from the index date to the date of the first cancer diagnosis, death, or the end of follow‐up (December 31, 2018), whichever came first. Hazard ratios (HRs) and 95% confidence intervals (CIs) for predicting the risk of cancer incidence and mortality in the ALD group were computed using Cox proportional hazards regression models. Because of greedy matching, there was a small proportion of differences between subgroups of matching variables (approximately 0.1% differences between cases and controls, see details in Table 1). In addition, a matched design could still introduce confounding, which requires controlling for the matching factors in the analysis.^33^ Therefore, in the multivariate model, we adjusted for all covariates. The results of the multivariate model were similar to those of the age‐adjusted model; therefore, they presented consistency. For liver cancer, chronic hepatitis B and C (ICD‐10: B18) was additionally adjusted for in the multivariate models. Multivariate analyses for the impact of ALD on the risk of cancer incidence and mortality according to hospital visit‐related variables were also performed, in which the linear trend *p*‐values were calculated using these variables as continuous variables. In the sensitivity analysis, the association between ALD and cancer risk was measured separately for hepatitis B and hepatitis C infections. Furthermore, odds ratios (ORs) and CIs calculated using logistic regression were assessed to determine the association between alcohol consumption and ALD, adjusted for age, income, smoking status, physical activity, and BMI. All statistical analyses were performed using SAS version 9.4 (SAS Institute, Inc.), and figures were visualized using R Studio 4.0.3 (RStudio, PBC). A *p*‐value below 0.05 was considered statistically significant.

**TABLE 1:** Characteristics of the study population by sex.

	Men				Women			
	Control (*n* = 833,748)		Case (*n* = 208,437)		Control (*n* = 80,320)		Case (*n* = 20,080)	
	*n*	%	*n*	%	*n*	%	*n*	%
Age, mean (SD)	48.32 (11.57)		48.41 (11.50)		51.06 (12.35)		51.09 (12.27)	
Income group								
Missing	19,204	2.30	4882	2.34	1034	1.29	230	1.15
Q1	109,526	13.14	25,983	12.47	19,163	23.86	5246	26.13
Q2	149,801	17.97	39,921	19.15	16,963	21.12	4677	23.29
Q3	237,007	28.43	63,080	30.26	17,803	22.17	4627	23.04
Q4	318,210	38.17	74,571	35.78	25,357	31.57	5300	26.39
Smoking status								
Never	297,724	35.71	74,371	35.68	73,706	91.77	18,420	91.73
Ex‐smoker	138,870	16.66	34,640	16.62	1353	1.68	347	1.73
Current smoker	397,154	47.63	99,426	47.70	5261	6.55	1313	6.54
Average alcohol consumption per day(gram/day)								
<5	197,696	23.71	49,424	23.71	62,784	78.17	15,696	78.17
5–9.9	137,968	16.55	34,492	16.55	7794	9.70	1948	9.70
10–19.9	211,693	25.39	52,920	25.39	5145	6.41	1283	6.39
20–29.9	125,404	15.04	31,027	14.89	2562	3.19	642	3.20
≥30	160,987	19.31	40,574	19.47	2035	2.53	511	2.54
Body Mass Index (kg/m^2^)								
Underweight or normal (<23)	267,374	32.07	68,041	32.64	33,771	42.05	8451	42.09
Overweight (23–24.9)	227,445	27.28	56,747	27.23	20,315	25.29	5072	25.26
Obesity (25+)	338,929	40.65	83,649	40.13	26,234	32.66	6557	32.65
Frequency of physical activity per week								
Missing	10,988	1.32	2904	1.39	987	1.23	273	1.36
No	366,626	43.97	101,041	48.48	49,131	61.17	12,713	63.31
1–2 times	278,725	33.43	64,671	31.03	15,584	19.40	3658	18.22
3–4 times	105,506	12.65	23,183	11.12	7598	9.46	1688	8.41
5–6 times	23,652	2.84	5105	2.45	2021	2.52	474	2.36
Everyday	48,251	5.79	11,533	5.53	4999	6.22	1274	6.34
Hypertension								
Missing	116	0.01	30	0.01	23	0.03	4	0.02
Normal	203,237	24.38	49,886	23.93	32,882	40.94	8125	40.46
Pre‐hypertension	433,796	52.03	110,573	53.05	33,002	41.09	8482	42.24
Hypertension	196,599	23.58	47,948	23.00	14,413	17.94	3469	17.28
Fasting blood sugar (mg/dl)								
Missing	517	0.06	161	0.08	67	0.08	21	0.10
Normal (<100)	547,926	65.72	129,671	62.21	60,363	75.15	14,491	72.17
Prediabetes (100–125)	219,271	26.30	60,116	28.84	15,972	19.89	4390	21.86
Diabetes (126+)	66,034	7.92	18,489	8.87	3918	4.88	1178	5.87
Total cholesterol (mg/dl)								
Missing	1057	0.13	299	0.14	132	0.16	35	0.17
Normal (<200)	464,917	55.76	114,228	54.80	43,300	53.91	10,670	53.14
Borderline high (200–239)	270,966	32.50	65,778	31.56	25,769	32.08	6,172	30.74
High (240+)	96,808	11.61	28,132	13.50	11,119	13.84	3203	15.95
CCI								
0	807,510	96.85	187,088	89.76	77,418	96.39	18,366	91.46
≥1	26,243	3.14	21,356	10.23	2902	3.62	1714	8.52

## 3 RESULTS

In our matched cohort study, 208,437 men and 20,080 women with ALD were matched in a 1:4 ratio for age, alcohol consumption, smoking status, and BMI group with 833,748 men and 80,320 women without ALD. The mean age was 48 and 51 years for men and women, respectively. Approximately 47.7% of men were current smokers, 19% consumed an average of at least 30 g of ethanol per day, 40% were obese, and nearly half did not exercise. Among the women, approximately 6.5% were current smokers, 78.2% consumed an average of fewer than 5 g of ethanol per day, 32.7% were obese, and more than 60% did not exercise. In both men and women, the ALD group had a higher proportion of individuals with diabetes, a high total cholesterol level, and comorbidities than the control group. Table 1 presents the detailed sociodemographic characteristics, lifestyle behaviors, and clinical characteristics of the study participants.

In men, 78,158 (9.37%) and 22,547 (10.82%) men developed cancer in the control and ALD groups, respectively. After adjusting for covariates, ALD increased the risk of development of all cancers by 1.23‐fold (95% CI = 1.22–1.26) compared with the control group. In terms of specific cancer sites, ALD was associated with a significantly higher risk of 10 cancer types, including lip, oral cavity, and pharynx; esophagus; liver; gallbladder and biliary tract; pancreas; larynx; lung; kidney; thyroid gland; and leukemia. In contrast, ALD was associated with a decreased risk of colorectal cancer development. In women, 6286 (7.83%) and 1708 (8.51%) subjects developed cancer in the control and ALD groups, respectively. The result was similar to that of men in that ALD significantly increased the risk of all cancers (HR = 1.12, 95% CI = 1.06–1.18), and other specific cancers of the esophagus and liver (Table 2).

**TABLE 2:** HRs and 95% CIs for the association between ALD and cancer incidence in both sexes

	Men				Women			
	Control (*n* = 833,748)	Case (*n* = 208,437)	Age adjusted	Fully adjusted	Control (*n* = 80,320)	Case (*n* = 20,080)	Age adjusted	Fully adjusted
	No. event (%)	No. event (%)	HR (95%CI)	HR (95%CI)	No. event (%)	No. event (%)	HR (95%CI)	HR (95%CI)
All cancers	78,158 (9.37)	22,547 (10.82)	**1.24 (1.22–1.26)**	**1.23 (1.21–1.25)**	6286 (7.83)	1708 (8.51)	**1.12 (1.06–1.18)**	**1.12 (1.06–1.18)**
Lip, oral cavity, and pharynx	1359 (0.16)	492 (0.24)	**1.55 (1.40–1.72)**	**1.49 (1.34–1.66)**	73 (0.09)	21 (0.10)	1.18 (0.73–1.92)	1.20 (0.73–1.95)
Esophagus	1690 (0.20)	730 (0.35)	**1.87 (1.72–2.04)**	**1.84 (1.68–2.01)**	10 (0.01)	9 (0.04)	**3.74 (1.52–9.21)**	**3.94 (1.59–9.76)**
Stomach	16,689 (2.00)	3991 (1.91)	1.02 (0.99–1.06)	1.03 (0.99–1.07)	687 (0.86)	172 (0.86)	1.03 (0.87–1.22)	1.03 (0.87–1.22)
Colon and rectum	12,271 (1.47)	2693 (1.29)	**0.94 (0.90–0.98)**	**0.95 (0.91–0.99)**	697 (0.87)	156 (0.78)	0.92 (0.78–1.10)	0.92 (0.77–1.09)
Liver	6095 (0.73)	4498 (2.16)	**3.15 (3.03–3.27)**	**2.34 (2.25–2.44)**	216 (0.27)	211 (1.05)	**4.04 (3.34–4.88)**	**2.60 (2.12–3.18)**
Gallbladder and biliary tract	1738 (0.21)	464 (0.22)	**1.17 (1.06–1.30)**	**1.18 (1.06–1.31)**	150 (0.19)	47 (0.23)	1.30 (0.94–1.81)	1.22 (0.87–1.70)
Pancreas	1949 (0.23)	541 (0.26)	**1.20 (1.09–1.32)**	**1.20 (1.09–1.33)**	181 (0.23)	39 (0.19)	0.90 (0.64–1.27)	0.85 (0.59–1.21)
Larynx	869 (0.10)	256 (0.12)	**1.27 (1.11–1.46)**	**1.28 (1.11–1.47)**	4 (<0.01)	1 (<0.01)	1.04 (0.12–9.30)	0.92 (0.10–8.76)
Lung	10,898 (1.31)	2832 (1.36)	**1.14 (1.09–1.19)**	**1.12 (1.07–1.17)**	447 (0.56)	101 (0.50)	0.94 (0.76–1.16)	0.93 (0.74–1.16)
Breast	_	_	_	_	1094 (1.36)	229 (1.14)	0.86 (0.74–0.99)	0.89 (0.77–1.02)
Cervix uteri	_	_	_	_	185 (0.23)	59 (0.29)	1.30 (0.97–1.75)	1.31 (0.97–1.76)
Corpus uteri	_	_	_	_	119 (0.15)	30 (0.15)	1.03 (0.69–1.54)	1.02 (0.67–1.54)
Ovary	_	_	_	_	125 (0.16)	35 (0.17)	1.15 (0.79–1.67)	1.08 (0.73–1.59)
Prostate	7759 (0.93)	1797 (0.86)	1.02 (0.97–1.08)	1.04 (0.98–1.09)	_	_	_	_
Testis	74 (0.01)	19 (0.01)	1.07 (0.65–1.78)	1.08 (0.64–1.81)	_	_	_	_
Kidney	1882 (0.23)	514 (0.25)	**1.16 (1.05–1.28)**	**1.17 (1.06–1.30)**	74 (0.09)	25 (0.12)	1.39 (0.88–2.19)	1.43 (0.91–2.25)
Bladder	2737 (0.33)	648 (0.31)	1.03 (0.94–1.12)	1.04 (0.95–1.13)	55 (0.07)	10 (0.05)	0.76 (0.39–1.49)	0.80 (0.41–1.58)
Brain	701 (0.08)	172 (0.08)	1.04 (0.88–1.23)	0.99 (0.84–1.18)	84 (0.10)	24 (0.12)	1.18 (0.75–1.85)	1.17 (0.74–1.85)
Thyroid gland	4188 (0.50)	1204 (0.58)	**1.20 (1.12–1.28)**	**1.22 (1.14–1.30)**	1459 (1.82)	370 (1.84)	1.03 (0.92–1.16)	1.07 (0.95–1.20)
Hodgkin lymphoma	69 (0.01)	11 (0.01)	0.68 (0.36–1.28)	0.63 (0.32–1.22)	3 (<0.01)	0 (0.00)	NA	NA
Multiple myeloma and malignant plasma cell neoplasma	512 (0.06)	88 (0.04)	0.74 (0.59–0.93)	0.76 (0.61–0.96)	57 (0.07)	12 (0.06)	0.87 (0.47–1.63)	0.76 (0.39–1.49)
Leukemia	944 (0.11)	260 (0.12)	**1.18 (1.03–1.35)**	**1.17 (1.01–1.34)**	66 (0.08)	19 (0.09)	1.19 (0.71–1.98)	1.19 (0.71–1.99)
Non‐Hodgkin lymphoma	140 (0.02)	31 (0.01)	0.94 (0.64–1.39)	0.97 (0.65–1.45)	7 (0.01)	2 (0.01)	1.18 (0.24–5.66)	1.26 (0.26–6.10)

Bold values denote statistical significanceat the 95% confidence level.

In men, 25,717 (3.08%) and 8228 (3.95%) cancer deaths occurred in the control and case groups, respectively. The ALD group had a increased risk of cancer death at all sites: the lip, oral cavity, and pharynx; esophagus; liver; gallbladder and biliary tract; pancreas; larynx; and lung. However, it was associated with a lower mortality risk for stomach and colorectal cancer. In women, there was an elevated risk of death from all cancers and from liver cancer in the ALD group (Table 3). Subgroup analysis by hepatitis B and C infection showed an increased HR of all cancer incidences and mortality in the ALD group, regardless of hepatitis B or C infection status, in both sexes (Tables S1 and S2).

**TABLE 3:** HRs and 95% CIs for the association between ALD and cancer mortality in both sexes

	Men				Women			
	Control (*n* = 833,748)	Case (*n* = 208,437)	Age adjusted	Fully adjusted	Control (*n* = 80,320)	Case (*n* = 20,080)	Age adjusted	Fully adjusted
	No. event (%)	No. event (%)	HR (95%CI)	HR (95%CI)	No. event (%)	No. event (%)	HR (95%CI)	HR (95%CI)
All cancers	25,717 (3.08)	8228 (3.95)	**1.39 (1.36–1.42)**	**1.34 (1.31–1.38)**	1272 (1.58)	384 (1.91)	**1.25 (1.12–1.40)**	**1.21 (1.08–1.36)**
Lip, oral cavity, and pharynx	369 (0.04)	171 (0.08)	**2.00 (1.67–2.39)**	**1.83 (1.52–2.21)**	10 (0.01)	3 (0.01)	1.25 (0.34–4.54)	1.23 (0.34–4.48)
Esophagus	920 (0.11)	385 (0.18)	**1.81 (1.61–2.04)**	**1.78 (1.58–2.01)**	4 (<0.01)	3 (0.01)	3.09 (0.69–13.8)	3.56 (0.78–16.18)
Stomach	3335 (0.40)	687 (0.33)	**0.89 (0.82–0.97)**	**0.89 (0.82–0.97)**	143 (0.18)	26 (0.13)	0.75 (0.50–1.14)	0.74 (0.48–1.13)
Colon and rectum	2483 (0.30)	498 (0.24)	**0.87 (0.79–0.96)**	**0.86 (0.78–0.95)**	129 (0.16)	25 (0.12)	0.80 (0.52–1.23)	0.74 (0.48–1.16)
Liver	3761 (0.45)	2681 (1.29)	**3.05 (2.90–3.20)**	**2.32 (2.20–2.44)**	122 (0.15)	122 (0.61)	**4.16 (3.23–5.34)**	**2.74 (2.10–3.60)**
Gallbladder and biliary tract	1023 (0.12)	266 (0.13)	**1.14 (1.00–1.31)**	**1.17 (1.02–1.35)**	82 (0.10)	28 (0.14)	1.42 (0.93–2.18)	1.35 (0.87–2.09)
Pancreas	1582 (0.19)	430 (0.21)	**1.17 (1.05–1.30)**	**1.17 (1.05–1.30)**	138 (0.17)	31 (0.15)	0.93 (0.63–1.38)	0.87 (0.58–1.30)
Larynx	138 (0.02)	56 (0.03)	**1.81 (1.32–2.47)**	**1.72 (1.25–2.38)**	0 (0.00)	0 (0.00)	NA	NA
Lung	7573 (0.91)	1947 (0.93)	**1.13 (1.07–1.19)**	**1.10 (1.05–1.16)**	224 (0.28)	50 (0.25)	0.93 (0.68–1.26)	0.92 (0.67–1.26)
Breast	_	_	_	_	67 (0.08)	12 (0.06)	0.73 (0.40–1.36)	0.72 (0.38–1.36)
Cervix uteri	_	_	_	_	27 (0.03)	6 (0.03)	0.92 (0.38–2.22)	1.00 (0.41–2.44)
Corpus uteri	_	_	_	_	13 (0.02)	4 (0.02)	1.26 (0.41–3.87)	1.34 (0.44–4.13)
Ovary	_	_	_	_	44 (0.05)	13 (0.06)	1.22 (0.66–2.26)	1.30 (0.69–2.42)
Prostate	775 (0.09)	179 (0.09)	1.06 (0.90–1.25)	1.02 (0.87–1.21)	_	_	_	_
Testis	4 (<0.01)	0 (0.00)	NA	NA	_	_	_	_
Kidney	269 (0.03)	62 (0.03)	1.00 (0.76–1.32)	1.00 (0.76–1.33)	10 (0.01)	1 (<0.01)	0.42 (0.05–3.26)	0.40 (0.05–3.15)
Bladder	390 (0.05)	95 (0.05)	1.09 (0.87–1.37)	1.08 (0.86–1.37)	9 (0.01)	2 (0.01)	0.84 (0.18–3.91)	0.76 (0.16–3.76)
Brain	335 (0.04)	80 (0.04)	1.02 (0.80–1.30)	0.99 (0.77–1.28)	38 (0.05)	7 (0.03)	0.76 (0.34–1.71)	0.74 (0.33–1.66)
Thyroid gland	40 (<0.01)	6 (<0.01)	0.65 (0.28–1.53)	0.69 (0.29–1.65)	7 (0.01)	2 (0.01)	1.19 (0.25–5.74)	1.29 (0.26–6.40)
Hodgkin lymphoma	12 (<0.01)	4 (<0.01)	1.49 (0.48–4.61)	1.12 (0.31–4.01)	1 (<0.01)	0 (0.00)	NA	NA
Multiple myeloma and malignant plasma cell neoplasma	278 (0.03)	41 (0.02)	0.64 (0.46–0.89)	0.65 (0.47–0.90)	22 (0.03)	8 (0.04)	1.52 (0.68–3.40)	1.40 (0.59–3.30)
Leukemia	490 (0.06)	131 (0.06)	1.15 (0.95–1.39)	1.14 (0.94–1.40)	39 (0.05)	8 (0.04)	0.85 (0.40–1.81)	0.85 (0.39–1.82)
Non‐Hodgkin lymphoma	17 (<0.01)	6 (<0.01)	1.53 (0.60–3.88)	1.50 (0.59–3.83)	0 (0.00)	1 (<0.01)	NA	NA

Bold values denote statistical significanceat the 95% confidence level.

**FIGURE 2:**
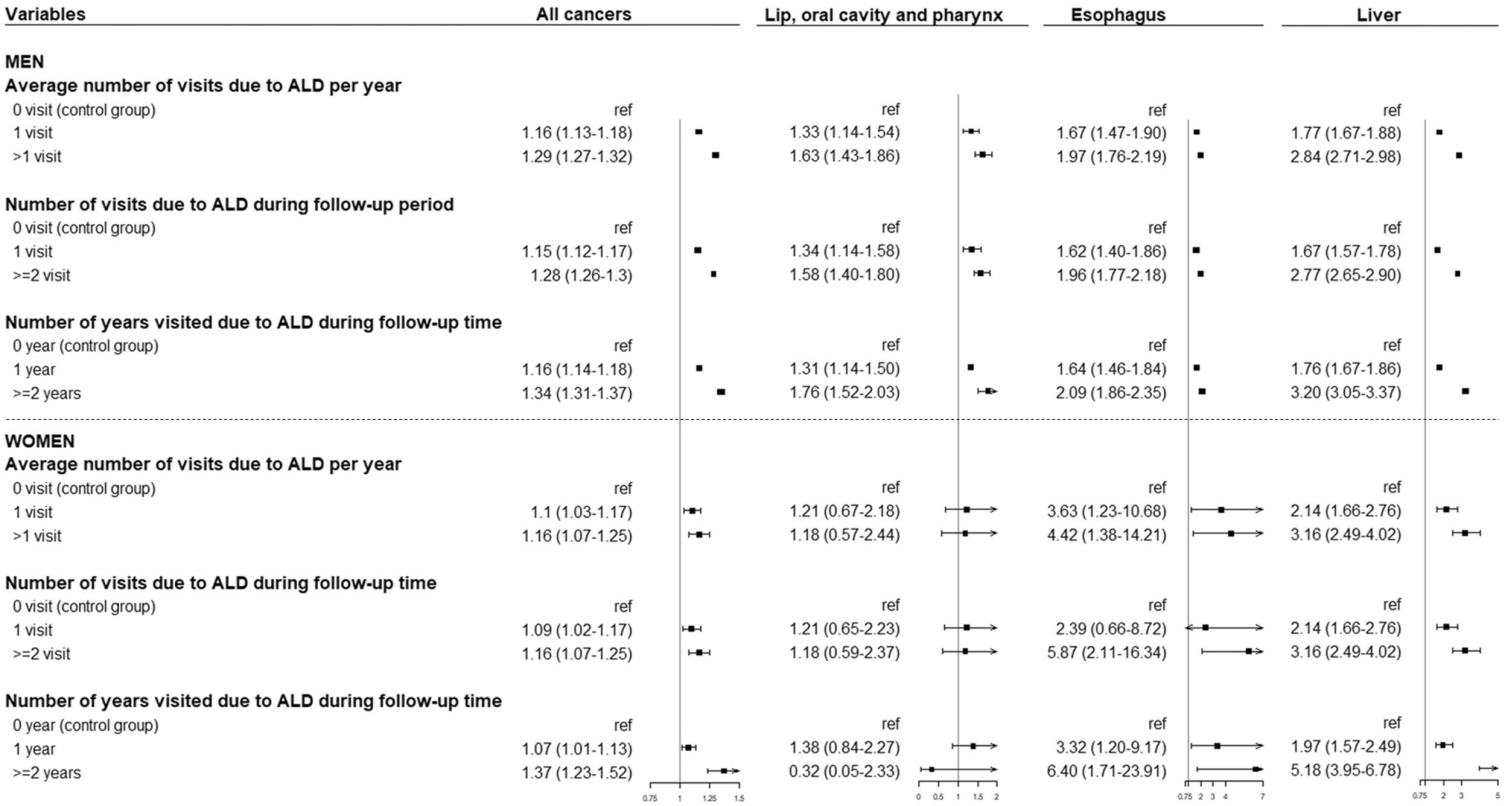
Forest plots for Cox proportional hazards model depicting the hazard ratios (95% confidence intervals) of ALD in relation to cancer incidence according to clinic or hospital visits.

**FIGURE 3:**
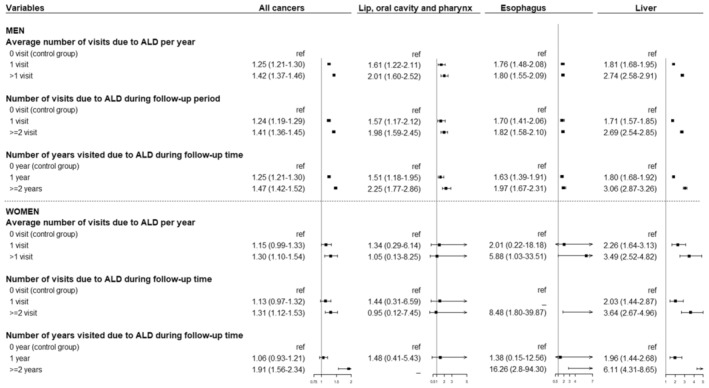
Forest plots for Cox proportional hazards model depicting the hazard ratios (95% confidence intervals) of ALD in relation to cancer mortality according to clinic or hospital visits.

Figures 2 and 3 show the association between ALD and cancer risk according to clinic or hospital visits. The results revealed that the more hospital visits for ALD, the higher the HR for the development of all cancers, esophageal cancer, and liver cancer in both sexes, and cancer of the lip, oral cavity, and pharynx in men only (*p*‐value for linear trend <0.001) (Figure 2). Similar findings were observed for cancer‐related mortality. In both men and women, a higher risk of death from all cancers and liver cancer was observed in ALD cases with a higher number of years of hospital visits (*p*‐value for linear trend <0.001) (Figure 3).

We also examined alcohol consumption in relation to ALD (Figure S2). We observed a clear dose–response relationship between average alcohol consumption and ALD. In both men and women, the more alcohol that was consumed, the higher the risk of ALD (*p*‐value for linear trend <0.001). Furthermore, the risk of developing ALD with similar alcohol consumption was higher in women than in men. In particular, exposure to an average of at least 30 g of ethanol daily elevated the risk by 5.1 times and 8.8 times in men and women, respectively, compared with those who consumed less than 5 g of ethanol daily.

## 4 DISCUSSION

This is the first study in Asia that includes a large number of patients with ALD to examine its relation to cancer incidence and mortality. To date, few studies have investigated the association between ALD and all‐cancer risk; the main focus has been on specific cancers, especially liver cancer. In 1998, the first study by Sørensen et al. in Denmark suggested a higher risk of various cancer types in patients with alcoholic cirrhosis.^6^ The risk of developing malignant neoplasms was high, yielding a standardized incidence ratio (SIR) of 2.2 (95% CI: 2.1–2.4). In a later study published in 2003, Sørensen et al. additionally showed findings on cancer risk in patients with fatty liver disease.^10^ They found that the overall risk of cancer was increased by 1.9‐fold (95% CI: 1.7–2.1) for patients with alcoholic fatty liver disease. In 2008, another study in England showed a significant 2.4‐fold risk increase for all cancers in individuals admitted to hospital with alcoholic cirrhosis (95% CI: 1.97–2.96).^7^ Furthermore, an SIR of 3.37 (95% CI: 3.13–3.61) was observed in Finnish patients with alcoholic cirrhosis.^8^ However, these studies did not consider the confounding effect of covariates—including alcohol consumption, smoking, and BMI—in the analysis. Given this limitation, we created a matched control group similar to the ALD group for several important variables, including age, sex, smoking, alcohol consumption, and BMI. We also adjusted for potential confounders (lifestyle behaviors and disease‐related variables) in our models. Our study found that ALD significantly increased by 1.23‐fold (95% CI: 1.21–1.25) and 1.12‐fold (95% CI: 1.06–1.18) the risk of all‐cancer incidence in men and women, respectively. Furthermore, we estimated the cancer mortality risk in the ALD group, which was not considered in the previous studies. In terms of all‐cancer mortality, our results suggested a 1.34‐fold higher risk in men (95% CI: 1.31–1.38) and a 1.21‐fold higher risk in women (95% CI: 1.08–1.36) in the ALD group relative to the control group. Given the insufficient population‐based epidemiological data on ALD globally, especially in Asia, our study provides novel findings to fill the knowledge gap for appropriately targeting clinical surveillance and the early detection of cancer.

An association between ALD and liver cancer is evident. ALD accounted for 30% of hepatocellular carcinoma (HCC) cases and HCC‐specific deaths worldwide.^5^ A matched cohort study that included 3410 patients with ALD showed an elevated risk of liver cancer with a sub‐distribution HR of 12.8 (95% CI: 9.38–17.45); however, again not adjusting for important cancer‐related confounders such as smoking or obesity status.^11^ Our study confirmed that ALD yielded more than a twofold risk of liver cancer incidence and mortality in both sexes compared with the control group. Besides the development of cirrhosis, which is considered a precancerous condition, there are several pathways in which alcohol induces liver carcinogenesis, including the formation of acetaldehyde and reactive oxygen species, changes to the immune system and the induction of chronic inflammation, and alterations to gene expression.^5^ Given the high annual incidence of liver cancer in patients with ALD, an HCC surveillance program plays a crucial role and should be recommended to detect HCC as early as possible in these patients. Furthermore, there is evidence showing that the implementation of an HCC surveillance program enables the promotion of early stage diagnosis and curative treatment, and increases overall survival.^5^, ^34^ In Korea, the nationwide liver cancer screening program was initiated in 2003,^35^ in which people aged 40 years or older with hepatitis B or C, liver cirrhosis, or chronic diseases of any cause (i.e., the high‐risk group) were eligible to participate.^36^ The program's cost‐effectiveness and beneficial impact on the mortality rate have been proposed in high‐risk groups;^36^ therefore, promising outcomes in terms of screening programs for patients with ALD can be anticipated.

Given that ALD develops due to long‐term alcohol exposure and this behavior can change over time, we calculated the mean amount of alcohol consumed during the entire look‐back period; the dose–response relationship between alcohol consumption and ALD was clear, and is consistent with the findings of previous studies.^37^ In fact, the alcohol consumption rate of the Korean population has increased over the past four decades,^19^ and has recently reached a distressing level.^38^ Moreover, there has been a significant decrease in chronic hepatitis B, whereas ALD cases have significantly increased.^21^, ^39^ Therefore, owing to the increased alcohol consumption and the fact that alcohol accounts for 18.6% of ALD and 6.8% of HCC^19^ cases, cancer‐related burdens may be exacerbated in the near future if alcohol control policies remain insufficient.^3^ Apart from liver cancer, this study also confirmed a strong association between ALD and the incidence and mortality of various other alcohol‐related cancers among men, including lip, oral cavity, and pharyngeal; esophageal; and laryngeal cancers. This finding is consistent with those of previous studies; in particular, there was a significantly higher risk of these cancers in patients with alcoholic cirrhosis,^6^, ^7^, ^8^ and alcoholic fatty liver disease.^10^ Meanwhile, in women, we found a 3.9‐fold increase of the development of esophageal cancer exclusively. The small number of observations may have affected other associations noted in women. In addition, in no prior studies were subgroup analyses performed by sex; therefore, we could not compare our findings with those of other studies.

Because we determined ALD cases using only the ICD‐10 code, the severity of ALD could not be measured. However, we used the number of clinic or hospital visits as an indirect index reflecting ALD severity. Our findings highlighted that the more times a patient visited a clinic or hospital for ALD, the higher the risk of cancer at all sites, including that of the liver, esophagus, lip, oral cavity, and pharynx (*p*‐‍value for trend <0.001). Thus, the need for a surveillance program targeting high‐risk patients with ALD who exhibit a high frequency of hospital visits should be emphasized.

Furthermore, evidence regarding the relationship between ALD and cancer at other sites is inconsistent. An elevated risk of lung cancer was observed in the ALD group relative to the control group, similar to the findings of a prior study.^6^, ^7^, ^8^, ^10^ We found an association between gallbladder and biliary tract cancer and ALD, while other studies did not.^6^, ^10^ The findings from this study showed that there was a higher risk of pancreatic cancer in the ALD group than in the control group, which is consistent with the results of several previous studies,^7^, ^8^ but contrary to those of others.^6^, ^10^ A significant association between ALD and renal cancer incidence has been documented in earlier studies^6^, ^8^ and in this study; however, another study showed no association.^10^ These inconsistent results could be due to the differences in study designs, sample sizes, and characteristics of the study samples. As mentioned above, no earlier studies created a control group and considered the confounding effects of other covariates. In addition, the large number of patients with ALD that was included in our study could guarantee sufficient statistical power (as reflected in the small CIs). Sharing the same risk factor (i.e., smoking) could explain the association of ALD with the cancer outcomes in this study; however, we controlled for smoking status by means of matching between the ALD and control groups. Moreover, the possible mechanism of ALD in the development of these cancer outcomes has not yet been explained in previous studies. Nevertheless, one explanation could be that comorbidities may contribute to the increased cancer risk in patients with ALD, as a higher proportion of patients with comorbidities was seen in the ALD group relative to the control group.

An increased risk of colorectal cancer in patients with ALD has been suggested in several previous studies given that alcohol consumption is common among patients with these cancers^6^, ^7^, ^8^, ^10^; however, our study revealed that ALD had a protective effect on colorectal cancer. This finding could be explained by the fact that the average age of a colorectal cancer diagnosis (mean: 56 years) was higher than that of an ALD diagnosis (mean: 48 years), and that ALD is associated with a low survival rate,^40^ thereby diminishing the development and risk of death from colorectal cancer. In particular, our study also supported this assumption that the mean age of all‐cause deaths was 58,86 years (median: 60 years), lower than the mean age of colorectal‐cancer deaths of 60,64 years (median: 62 years) among ALD patients. Another proposed explanation is that patients with ALD are seemingly more likely to undergo health check‐ups, including colorectal cancer screening, than the general population, which could reduce the risk of death from this cancer and also explain the lower risk of death from stomach cancer. Furthermore, eating behaviors—such as consuming a high amount of salt, smoked foods, or red meat—comprise the main risk factors for stomach and colorectal cancer, which may mediate the association between ALD and cancer outcomes. However, we did not have nutritional data or other relevant variables to adjust for in our models, which could have confounded our results.

Although this study has several advantages over previous studies, it still has some limitations. First, due to changing the general health examination questionnaire in 2009, we included ALD cases from 2002 to 2008. The number of ALD cases was relatively smaller in women than men, leading to a limited number of incidents and deaths in rare cancers, which could affect statistical power for these cancers in the survival analysis. However, the large number of patients with ALD included in our study is much higher than that used in previous studies. Second, although the case and control groups were matched by alcohol consumption, the amount of alcohol consumed before 2002 was not captured, which could have confounded our findings. Third, other important confounding variables on nutrition and eating behavior could not be obtained in our study. Nevertheless, we have included several other essential factors such as alcohol consumption, smoking, BMI group, physical activity, and comorbidities that other studies did not. Finally, there is lack of an additional set of covariates significantly developing specific cancer sites (e.g., *H. pylori* infection in relation to gastric cancer), which could possibly overestimate the association of ALD with cancer risk. However, *H. pylori* infection is highly prevalent in Korea (seroprevalence: 59.6% in 2005).^41^ Given no association between *H. pylori* infection and ALD suggested in a large study in China,^42^ but no evidence in South Korea, *H. pylori* is not likely a confounding factor in the estimation of the effect of ALD.

## 5 CONCLUSION

Patients with ALD had an elevated risk of all‐cancer and liver cancer incidence and mortality compared to the matched control group. In men, we also observed a higher risk of the development of and death from cancer at various sites, including the lip, oral cavity, and pharynx; esophagus; larynx; gallbladder and biliary tract; pancreas; and lungs. Additionally, we observed a dose–response relationship between ALD and alcohol consumption, which was more profound in women than in men. Given the lack of population‐based epidemiological data on ALD in Asia, our study could provide novel evidence to fill the knowledge gap for appropriately targeting clinical surveillance and the early detection of cancer in patients with ALD.

## AUTHOR CONTRIBUTIONS

**Thi Phuong Thao Tran:** Conceptualization (equal); formal analysis (equal); writing – original draft (equal); writing – review and editing (equal). **Minji Han:** Conceptualization (equal); formal analysis (equal); resources (equal); writing – original draft (equal); writing – review and editing (equal). **Ngoc Minh Luu:** Formal analysis (equal); writing – review and editing (equal). **Jin‐Kyoung Oh:** Conceptualization (equal); resources (equal); supervision (equal); writing – review and editing (equal).

## FUNDING INFORMATION

This work was supported by the National Cancer Center (grant number NCC‐2010303). The funder had no role in study design, data collection and analysis, decision to publish, or preparation of the manuscript.

## CONFLICT OF INTEREST

The authors declare no competing interests.

## ETHICS STATEMENT

As this study used anonymous secondary data, the study was exempted from review by the Institutional Review Board of the National Cancer Center, Korea (NCC2018‐0279). This study was conducted according to the Declaration of Helsinki.

## Supporting information


Figure S1

Figure S2

Table S1

## Data Availability

The datasets generated and analyzed during the current study (NHIS‐2020‐1‐237) cannot be shared because NHIS prohibits the transfer, rental, or sale of the database to third parties except for researchers who have been approved for access. NHIS data are available upon request from the National Health Insurance Sharing Service, https://nhiss.nhis.or.kr/.
