# Supplementary figures and images for: Alcoholic liver disease in relation to cancer incidence and mortality: Findings from a large, matched cohort study in South Korea
Source: Cancer Med. 2023 Jan 18;12(7):8754–66. doi: 10.1002/cam4.5614 (PMC10134281; doi:10.1002/cam4.5614)

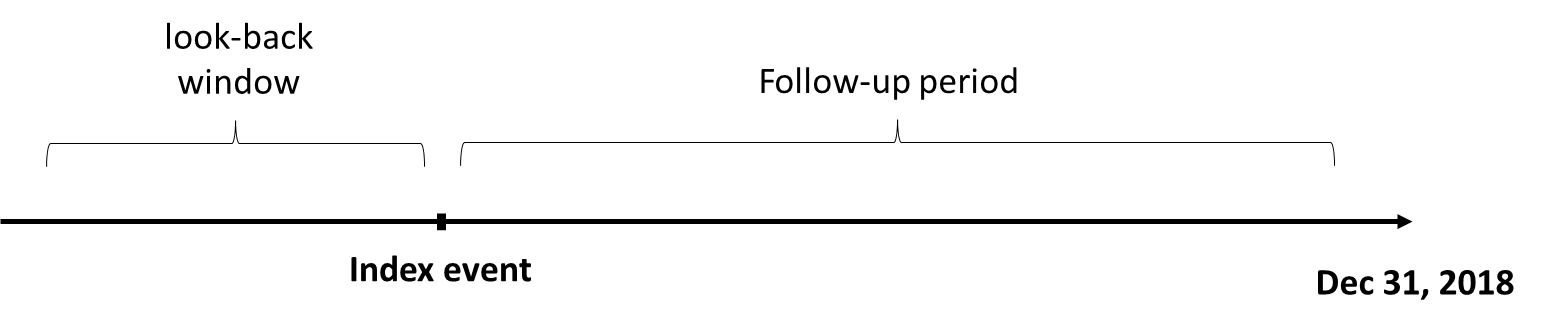

Supplement: Supplementary file 1 — Figure S1 [file CAM4-12-8754-s002.tif]

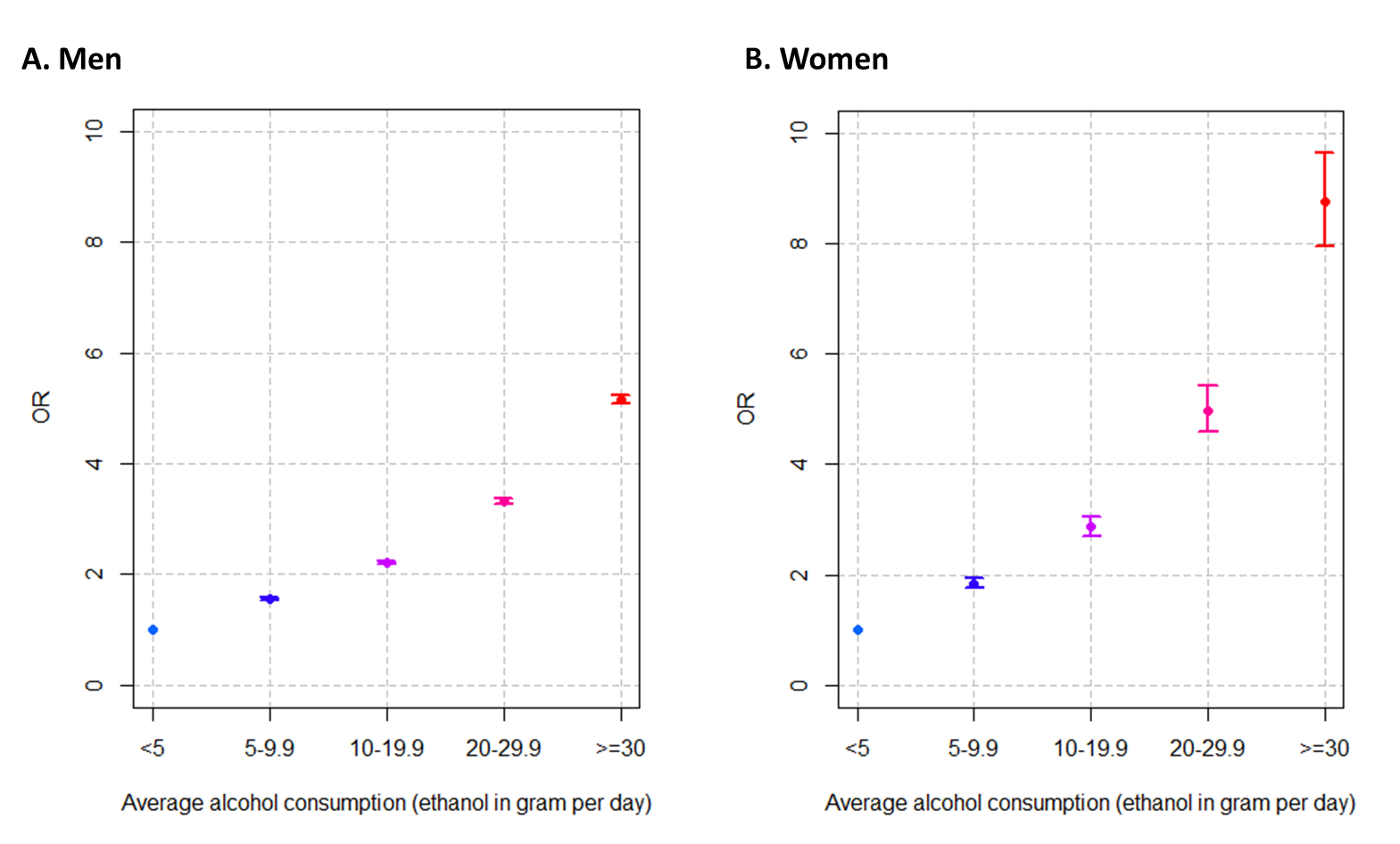

Supplement: Supplementary file 2 — Figure S2 [file CAM4-12-8754-s001.tif]
