# Supplementary material for: Alcoholic liver disease in relation to cancer incidence and mortality: Findings from a large, matched cohort study in South Korea
Source: Cancer Med. 2023 Jan 18;12(7):8754–66. doi: 10.1002/cam4.5614 (PMC10134281; doi:10.1002/cam4.5614)
Supplement: Supplementary file 3 — Table S1 [file CAM4-12-8754-s003.docx]

**Table S1: HRs and 95% CIs for the association between ALD and cancer incidence in both sexes, by HBV/HCV status**

|  | **Men** | | **Women** | |
| --- | --- | --- | --- | --- |
|  | Infection with HCV/HBV | No infection with HCV/HBV | Infection with HCV/HBV=1 | No infection with HCV/HBV=0 |
| All cancers | **1.10 (1.03-1.17)** | **1.20 (1.18-1.22)** | **1.27 (1.01-1.59)** | **1.08 (1.02-1.14)** |
| Lip, oral cavity and pharynx | 1.14 (0.66-1.96) | 1.49 (1.34-1.66) | 0.32 (0.02-4.08) | 1.23 (0.75-2.03) |
| Esophagus | 1.33 (0.74-2.38) | 1.86 (1.70-2.04) | NA | 3.64 (1.43-9.27) |
| Stomach | 1.00 (0.82-1.22) | 1.03 (0.99-1.07) | 1.00 (0.43-2.28) | 1.01 (0.85-1.21) |
| colon and rectum | 0.88 (0.69-1.11) | 0.95 (0.91-1.00) | 0.59 (0.23-1.48) | 0.93 (0.77-1.11) |
| Liver | **1.13 (1.03-1.23)** | **2.87 (2.75-3.01)** | **1.68 (1.16-2.42)** | **3.10 (2.45-3.92)** |
| Gallbladder and biliary tract | 1.19 (0.66-2.14) | 1.18 (1.06-1.31) | NA | 1.22 (0.86-1.71) |
| Pancreas | 1.34 (0.80-2.23) | 1.19 (1.08-1.32) | 0.57 (0.04-8.69) | 0.87 (0.61-1.25) |
| Larynx | 2.34 (1.08-5.07) | 1.24 (1.07-1.43) | NA | 0.96 (0.10-9.13) |
| Lung | 1.19 (0.93-1.51) | 1.11 (1.07-1.16) | 0.49 (0.15-1.62) | 0.95 (0.76-1.19) |
| Breast | _ | _ | 0.92 (0.42-1.99) | 0.89 (0.77-1.03) |
| Cervix uteri | _ | _ | 1.60 (0.36-7.09) | 1.28 (0.94-1.74) |
| Corpus uteri | _ | _ | 0.54 (0.09-3.18) | 1.02 (0.67-1.57) |
| Ovary | _ | _ | NA | 1.09 (0.73-1.62) |
| Prostate | 0.84 (0.63-1.10) | 1.04 (0.99-1.10) | _ | _ |
| Testis | NA | 1.16 (0.69-1.96) | _ | _ |
| Kidney | 1.09 (0.68-1.74) | 1.17 (1.05-1.29) | 0.77 (0.12-5.1) | 1.44 (0.90-2.31) |
| Bladder | 1.22 (0.70-2.11) | 1.04 (0.95-1.14) | NA | 0.66 (0.32-1.40) |
| Brain | 0.84 (0.34-2.07) | 1.00 (0.84-1.20) | NA | 1.09 (0.67-1.76) |
| Thyroid gland | 1.15 (0.86-1.53) | 1.21 (1.13-1.29) | 1.28 (0.73-2.25) | 1.06 (0.94-1.19) |
| Hodgkin lymphoma | NA | 0.65 (0.33-1.27) | NA | NA |
| Multiple myeloma and malignant plasma cell neoplasma | 0.89 (0.36-2.24) | 0.74 (0.58-0.93) | NA | 0.79 (0.40-1.56) |
| Leukemia | 0.91 (0.43-1.92) | 1.18 (1.02-1.36) | NA | 0.98 (0.56-1.72) |
| Non-Hodgkin lymphoma | 4.50 (0.45-44.54) | 0.91 (0.60-1.38) | NA | 1.32 (0.27-6.38) |

**Table S2: HRs and 95% CIs for the association between ALD and cancer mortality in both sexes, by HBV/HCV status**

|  | **Men** | | **Women** | |
| --- | --- | --- | --- | --- |
|  | Infection with HCV/HBV | No infection with HCV/HBV | Infection with HCV/HBV | No infection with HCV/HBV |
| All cancers | **1.24 (1.13-1.37)** | **1.29 (1.26-1.33)** | **1.62 (1.03-2.52)** | **1.11 (0.98-1.26)** |
| Lip, oral cavity and pharynx | 2.16 (0.54-8.64) | 1.84 (1.52-2.23) | NA | 1.27 (0.35-4.65) |
| Esophagus | 1.89 (0.70-5.09) | 1.81 (1.59-2.04) | NA | 3.64 (0.8-16.57) |
| Stomach | 1.15 (0.67-1.98) | 0.89 (0.82-0.97) | NA | 0.71 (0.46-1.11) |
| colon and rectum | 0.74 (0.4-1.35) | 0.87 (0.79-0.97) | NA | 0.74 (0.47-1.16) |
| Liver | **1.26 (1.12-1.42)** | **2.65 (2.50-2.80)** | **1.93 (1.14-3.27)** | **3.08 (2.28-4.17)** |
| Gallbladder and biliary tract | 1.58 (0.66-3.82) | 1.18 (1.03-1.36) | NA | 1.36 (0.87-2.12) |
| Pancreas | 1.30 (0.74-2.28) | 1.15 (1.03-1.29) | 0.46 (0.01-20.55) | 0.88 (0.59-1.32) |
| Larynx | 2.09 (0.32-13.55) | 1.71 (1.23-2.38) | NA | NA |
| Lung | 1.22 (0.90-1.64) | 1.10 (1.04-1.16) | 0.38 (0.04-3.86) | 0.95 (0.69-1.31) |
| Breast | _ | _ | NA | 0.76 (0.40-1.45) |
| Cervix uteri | _ | _ | NA | 1.04 (0.43-2.54) |
| Corpus uteri | _ | _ | NA | 1.51 (0.49-4.69) |
| Ovary | _ | _ | NA | 1.35 (0.72-2.52) |
| Prostate | 1.06 (0.38-2.96) | 1.03 (0.87-1.22) | _ | _ |
| Testis | NA | NA | _ | _ |
| Kidney | 2.24 (0.36-13.89) | 1.00 (0.75-1.33) | NA | 0.41 (0.05-3.24) |
| Bladder | 5.04 (0.23-110.73) | 1.10 (0.87-1.38) | NA | 0.79 (0.16-3.89) |
| Brain | 0.22 (0.03-1.93) | 1.03 (0.80-1.33) | NA | 0.67 (0.28-1.59) |
| Thyroid gland | NA | 0.72 (0.30-1.71) | NA | 1.36 (0.27-6.78) |
| Hodgkin lymphoma | NA | 1.15 (0.32-4.13) | NA | NA |
| Multiple myeloma and malignant plasma cell neoplasma | 0.66 (0.22-1.99) | 0.61 (0.43-0.87) | NA | 1.45 (0.62-3.43) |
| Leukemia | 1.05 (0.35-3.16) | 1.15 (0.94-1.41) | NA | 0.67 (0.28-1.59) |
| Non-Hodgkin lymphoma | NA | 1.55 (0.61-3.97) | NA | NA |
